# Embodiment as a Paradigm for Understanding and Treating SE-AN: Locating the Self in Culture

**DOI:** 10.3389/fpsyt.2020.00534

**Published:** 2020-06-12

**Authors:** Connie Marguerite Musolino, Megan Warin, Peter Gilchrist

**Affiliations:** ^1^College of Medicine and Public Health, Southgate Institute for Health, Society and Equity, Flinders University, Bedford Park, SA, Australia; ^2^Faculty of Arts, School of Social Sciences, University of Adelaide, Adelaide, SA, Australia; ^3^Private Practitioner, Adelaide, SA, Australia

**Keywords:** Severe and Enduring Anorexia Nervosa, embodiment, culture, *habitus*, qualitative, harm minimization, quality of life

## Abstract

There has been a growing call for sociologically engaged research to better understand the complex processes underpinning Severe and Enduring Anorexia Nervosa (SE-AN). Based on a qualitative study with women in Adelaide, South Australia who were reluctant to seek help for their disordered eating practices, this paper draws on anthropological concepts of embodiment to examine how SE-AN is experienced as culturally grounded. We argue that experiences of SE-AN are culturally informed, and in turn, inform bodily perception and practice in the world. Over time, everyday rituals and routines became part of participants’ *habitus’*, experienced as taken-for-granted practices that structured life-worlds. Here, culture and self are not separate, but intimately entangled in and through embodiment. Approaching SE-AN through a paradigm of embodiment has important implications for therapeutic models that attempt to move anorexia nervosa away from the body and separate it from the self in order to achieve recovery. Separating experiences—literally disembodying anorexia nervosa—was described by participants as more than the loss of an identity; it would dismantle their sense of being-in-the-world. Understanding how SE-AN is itself a structure that structures every aspect of daily life, helps us to understand the fear of living differently, and the safety that embodied routines bring. We conclude by asking what therapeutic treatment might look like if we took embodiment as one orientation to SE-AN, and focused on quality of life and harm minimization.

## Introduction

When in a rabbit hole, you become largely blind (or nonchalant) to those and the world around you. You are too far down to see them much or properly. And it is all too comfortable, familiar and interesting for you to care otherwise.

You also become deaf to any voice of opposition. What do they know? You know what you’re doing, you have things in hand. Go away, people, and just leave me to it.

And the older you get and the more times you’ve been in the rabbit holes, the more they do just leave you to it.

And for some reason, some small hidden part of you quietly wishes that they wouldn’t.

- Charlotte’s diary entry, 2^nd^ of April, 2013

This diary entry is from a 32-year-old Australian woman who has had severe disordered eating for more than half her life. Charlotte’s spatial metaphor of the “rabbit hole” captures the safety and comfort which her “dark home” affords. Having lived in her protective space for many years and not wanting to come out, she is also aware of how hard it is to leave. She has undertaken multiple therapeutic treatments (with minimal success), and each time she has been drawn back down into the familiar rabbit hole of disordered eating. A small part of her wishes people would keep trying to pull her out, but like so many people with enduring eating disorders, her relationship with recovery and help seeking remains highly ambivalent.

While consensus concerning the diagnostic criteria for Severe and Enduring Anorexia Nervosa (SE-AN) remains contentious (1–4), and there are inconsistencies in the definition and labelling of SE-AN in the medical and psychiatric literature (1, 4–7), Charlotte’s experiences align with the chronic, severe and enduring nature of this diagnosis. When we met Charlotte in 2013, she had had a previous diagnosis of anorexia nervosa in her late teens, and self-reported 15 plus years of significant eating disorder behaviors. During our research, she admitted herself to a psychiatric service for in-patient care, and otherwise remained living in the community, self-managing myriad, everyday negotiations around food and eating. Charlotte’s length of illness duration, her number of previously failed treatment attempts, resistance to traditional treatments, and entrenched and persistent patterns of behavior, correspond to Broomfield et al.’s (1) identification of the most common defining features of SE-AN.

Due to the severity and chronicity of SE-AN, it is not surprising that there is uncertainty around the long-term outlook and whether recovery is possible for everyone. Broomfield et al. (1) note that the term “chronic” (which often implies an incurable illness) is currently the most commonly used adjective in the literature to describe SE-AN. Touyz and Hay (6) certainly argue that most patients with SE-AN are unlikely to fully recover (as defined by clinical criteria), and this is supported in the growing number of long-term clinical studies (8).

There are concerns that current mainstream treatment approaches to SE-AN exacerbate suffering and feelings of failure, and increase resistance to seeking help (1, 5, 9–11). Therefore, many clinicians working with this group over the last decade have been turning to more holistic biopsychosocial approaches to therapeutic care that go beyond symptom reduction, such as harm minimization approaches, and focusing on quality of life and improving general everyday functioning (1, 6, 8, 12–14).

In seeking to understand what quality of life might mean to people with a diagnosis of SE-AN, clinicians and researchers have recently called for more “sociologically engaged” research (6, 15). A number of psychiatric and medical studies have attempted to do this by using traditional qualitative methods of interviews to elevate the voices of people with disordered eating and their carers (13, 16–18). While these studies provide valuable information about people’s experiences (e.g., 13, 14, 19), they tend to use what anthropologists call “etic” or “outsider” perspectives, in this case understanding SE-AN through a lens of disease categories and individual psychopathology.

Anthropologists on the other hand, spend time with people in their own environments (often over many months of fieldwork), conducting participant observation and exploring how people navigate particular aspects of their daily lives. This is an explicit focus on “the emic” or insiders’ experience that allows the researcher to get close to complex experiences while remaining critical of their own bias. It is through this emic approach that anthropology seeks “to begin with and build upon [peoples] meanings and theories rather than their own” [(20), p.130].

Anthropology not only provides a rigorous and innovative methodological approach (beyond interviews) but seeks to identify the cultural structures that shape and inform mental health. This cultural orientation to mental illness attends to the “cultural making of forms of subjectivity [and intersubjectivity] from the actor’s point of view—where the question is how actors enact, resist, or negotiate the world as given, and in so doing, make the world” [(21), p. 9]. In other words, anthropological approaches interrogate the “cultural processes in constructing the experience of illness” [(21), p. 6], and how symptoms (and their meaning and interpretations) are, in turn, shaped by culture.

It is widely acknowledged that eating disorders have a substantial sociocultural component [see, for example, (22–28)], yet this dimension in clinical literature is often absent or reduced to stereotypes of “ethnicity” or “femininity” as a stand-in for culture. A recent edition of *Transcultural Psychiatry*—dedicated to anthropological approaches to eating disorders—demonstrates the value of culture with a series of research papers by anthropologists who examine the “cultural logics that drive eating disordered practices”, [showing] how these practices are embodied within everyday, normative milieus and broader cultural patterns [(29), p. 445].

In this paper, we draw on anthropological concepts of embodiment to examine SE-AN within sociocultural contexts, arguing that experiences of SE-AN are culturally informed, and in turn, inform bodily perception and practice in the world. In our study, we found that over time, everyday eating disorder rituals and routines that are enmeshed in cultural worlds became part of participants’ *habitus*, experienced as taken-for-granted practices that structured life-worlds. Here, culture and self are not separate, but intimately entangled in and through embodiment.

We begin the paper by outlining the anthropological concept of embodiment, and how this was developed as a critique of Enlightenment dualisms of mind/body. The anthropology of the body has a significant historical relationship with psychiatry, and our analysis begins with Csordas’ (30) concept of embodiment as this marked a turning point in theorizing bodies as being-in-the-world, of experience, perception, sentience, and practice (*habitus*). Following a description of the study, we present three key findings (safety, healthism, and structure) that demonstrate how participants’ experiences of anorexia nervosa were culturally situated and embodied. In framing participant experiences as the embodiment of *habitus*, the discussion section argues that these deeply held bodily dispositions are an embodiment of cultural practices. Taking an embodiment approach to SE-AN highlights how models of treatment and recovery which attempt to separate disordered eating from cultural practices and the “self”, not only operate within culturally constructed dualisms of the body and mind, but point directly to the profound ambivalence that this group has toward recovery. In the conclusion, we suggest that the paradigm of embodiment can inform harm minimization and quality of life approaches to care in SE-AN.

## The Embodiment of Cultural Practices

Building on a rich history of collaboration and conversation between psychiatry, philosophy, and anthropology (e.g., 31–37), our focus is on anthropological approaches that position cultural practices as central to experiences of bodies, selves, and everyday worlds.

Anthropologists have long been drawn to “the body” (and food), and classic ethnographic work (including cross-cultural research) has illuminated the ways in which understandings of the body vary according to differing historical and cultural contexts [for example, (31, 38–40)]. As a conceptual framing, embodiment came into anthropological writing as a specific critique of and departure from Cartesian dualism. This dominant Enlightenment thinking constructed knowledge as a series of key, hierarchical divisions between nature and culture, the biological and social, sex and gender, the individual and collective, and the body and mind. The mind and body were thus not only conceived as separate entities, but the body was understood to be a passive and fixed entity upon which society inscribed its rules. This ahistorical, acultural, and universal understanding of the body came to underpin the biomedical model in medicine (41).

In rejecting the mind/body dichotomy, Csordas argued that “the body is not an object to be studied in relation to culture, but is to be considered as the subject of culture, or in other words, as the existential ground of culture” [(30), p. 5]. The body is thus not an empirical “thing” that stands in as a backdrop to cultural life, but is an experiencing agent that is intersubjective, relational, dynamic, sentient, and indeterminate in nature (24). For Csordas (42), embodiment was a methodological and analytical tool that bridged dualisms.

Csordas’ paradigm of embodiment is key to articulating and theoretically framing the ways in which women with SE-AN in our study inhabited, transformed and reproduced a cultural “logic of practice”—what the French anthropologist Pierre Bourdieu refers to as the *habitus*. The *habitus* is a “system of lasting, transposable dispositions” that provide individuals with a sense of how to act and respond in the course of their everyday lives [(43), p. 95]. It underpins how we experience our bodies, for it is through the body that one learns the taken-for-granted rules of everyday life, such as accents, gestures, and preferences for food, fashion and entertainment [(44), p. 252]. It is through the *habitus* that cultural tastes for certain foods (fried chicken or caviar) and bodily dispositions are socially informed and embodied, in which girls in some cultures are taught to “eat like birds”, aspire to culturally constructed bodily ideals, and take up as little space as possible (“cross your legs”). Food and eating are much more than consumption of nutrients; they are deeply engrained in one’s *habitus*, displaying elaborate performances of cultural positionings—class distinctions, gender, taste, and identities.

In terms of eating disorders, we have previously argued that a “healthism *habitus*” (26)—the incessant pursuit of “good health” through a plethora of diets and fitness regimes—enables people with disordered eating to readily engage in taken-for-granted cultural norms of “health” that are embodied and structure social worlds. These eating disorder behaviors are not experienced as irrational or bizarre symptoms, but embodied as culturally shaped, highly gendered, and normative practices of health—in other words, part-and-parcel of one’s *habitus*.

It’s important to highlight that the words “habit” and *habitus* for Bourdieu are not the same; habit is a mechanical behavior, whereas “*habitus*” involves a flexible disposition that entails competence, skill, and know-how (what Bourdieu calls the collective repertoire of practical reason) (43, 45). *Habitus* foregrounds social dimensions that are culturally situated and practiced, whereas “habit” in eating disorder literature follows a different understanding and foregrounds individual compulsive behaviors and stimulus responses [see, for example, (46)].

## Methods

### Participants

This paper examines the experiences of a subgroup of women from a larger mixed methods study, including ethnographic fieldwork and psychological evaluation. The purpose of the study was to explore the cultural contexts of disordered eating among women in Adelaide, South Australia with the aim of understanding why they were reluctant to seek help. Data collection occurred over 15 months (January 2013 to March 2014) and involved 28 women ranging in age from 19 to 52. As the project was primarily interested in why women delay seeking help for disordered eating, the criteria for recruitment included women who were over 16 years of age who had not seen a health professional for disordered eating; had not been given an eating disorder diagnosis; had been diagnosed but had delayed seeking treatment; or who did not wish to pursue treatment. Following consent to take part in the Eating Disorder Examination [EDE; (47)], the majority (75%) of study participants met the criteria for a diagnosable eating disorder (48).

The recruitment criteria for the larger study were intentionally broad and we did not purposefully recruit women who fell into a SE-AN category. Here, we focus on five women in the study group (between 27 and 52 years of age) who had been diagnosed and living with anorexia nervosa for more than 10 years (see **Table 1** below). Two key factors contributed to this smaller group of women being retrospectively selected as potentially meeting the criteria for SE-AN out of the overall sample: 1) the five women who were selected had historically received a diagnosis of anorexia nervosa, whereas the other participants had not been clinically diagnosed prior to participating in this study. For the women without a prior diagnosis, it was difficult to establish severity and longevity of their disordered eating; 2) the overall sample were generally younger, ranging from 19 to 30 years of age, and had self-reported disordered eating from 6 months to 10 years. The number of women (5) we thus focus on is entirely consistent with those who develop SE-AN (20% of those diagnosed with anorexia nervosa [(49), p. 314]).

**Table 1:** Participant characteristics.

Participant	Age	Previous diagnosis	EDE Diagnostic results	Self-reported length of disordered eating
**Kelly**	40	AN	AN	20+ years
**Charlotte**	32	AN	Did not meet criteria.	15+ years
**Michelle**	27	AN	EDNOS—weight/BMI not recorded, otherwise psychopathology consistent with AN	10+ years
**Sarah**	28	AN	AN—Binge-eating/purging type	15+ years
**Lorraine**	52	AN	AN	30+ years

### Recruitment

Participants were recruited through purposeful sampling methods from two metropolitan university campuses in Adelaide, South Australia, through South Australian mental health networks and advertising on social media websites such as Facebook groups (South Australian Body Esteem Activists and Supporting Eating Disorders for South Australia). Ethnographic research methods are critical to accessing the everyday practices and private experiences of hard to reach groups such as the population in this study who often did not identify as having a problem, faced social stigma, and were reluctant or too overwhelmed by their situation to engage with services [see (26)]. Therefore, recruitment posters were placed on the backs of toilet doors and pin boards and posed questions such as “Are you continually thinking about your food and your weight?” and “Do you enjoy the feeling of not eating or excessive exercising?”

The study received approval from the University of Adelaide Human Research Ethics Committee (H-2012-069) and the Southern Adelaide Clinical Human Research Ethics Committee (SAC HREC EC00188). Prior to giving consent, all participants were fully informed about the research and the nature of their participation and understood they had the right to withdraw from the study at any time. In agreeing to participate in the study, participants were informed that information gained during the study may be published, and that they would be de-identified in all sources. Participants were provided with a copy of their signed consent form, along with information and resources on available eating disorder services.

### Data Collection

Author 1 conducted semi-structured interviews and participant observation in people’s homes, in interview rooms at one of the universities, in cafes and in public places. Two to three semi-structured interviews, plus the EDE, were conducted with each participant over a number of months. Due to the exploratory nature of qualitative research, the interview schedule was flexible, and interviews varied in length (averaging 1–2 hours). Field notes taken during and after interviews captured observations made during the research meetings, such as non-verbal cues, emotional reactions, appearances, the research setting, as well as conversations outside of the interviews not recorded. The familiar interview settings of homes and the multiple interviews with each participant allowed rapport to build between the researcher and participant and provided opportunities to explore their everyday lives in more detail, not otherwise accessible in clinical settings or in one-off interviews.

In terms of research rigour and our ethical responsibilities, the inclusion of the EDE was important to ascertain if participants might meet psychiatric classifications, to elicit their responses to such evaluations, and to provide them with information about resources and services. We recognize that the language associated with psychiatric diagnostic classifications have value in clinical contexts and the wider community (including policy and practice contexts), but equally that terms such as, “disorder”, “illness”, “health”, and “care” can have different meanings in other contexts, and this was important in engaging a population group reluctant to seek help. For some of the women in this subgroup, they did not identify with the words “illness” and “disorder”, despite receiving a clinical diagnosis, but also discussed reluctantly needing to use their diagnosis when accessing the health and social security systems. As the project aimed to explore the denial of eating issues and the delay in seeking treatment, it was not surprising that the women in this study had had varying contact with health providers, and were all resistant to recover (in medical terms).

In addition to the interviews, diary writing was embedded in the ethnographic phase at the end of the interviews for a period of 8 weeks. Participants were invited to record such things as “the everyday moments, activities or events that might support your eating disorder behaviors … your fears, pleasures and desires around food and your body—what are you scared of? What do you need and want”? They could “draw, doodle, write a lot, very little”. The collection of diaries also allowed for follow-up questions, including asking about their future help-seeking plans and a debrief of the study. Diaries are widely used in ethnography and social anthropology as a tool of inquiry, and are viewed as a “classic articulation of dailyness” [(50), p. 95]. It is this “everydayness” that the diary writing aimed to capture and helped to overcome problems associated with collecting sensitive information, allowing participants to jot down their thoughts or feelings as they were happening, providing insights into intimate eating and bodily activities.

### Analysis

The research design and methods were guided by grounded theory principles, coupled with thematic techniques of data collection and analysis (51–53). Grounded theory is a qualitative inductive methodology which prioritizes developing analytic categories and themes directly from the data, not from pre-conceived concepts or hypotheses, while also being reflexive of the bias that the researcher brings to the interpretation of the data (51). All interviews (including semi-structured and EDE interviews) were digitally recorded and professionally transcribed, and field notes were written up directly following each interview.

Following a process of open, axial and selective coding, the interview transcripts and field note data were firstly open coded in a Word document, and then through the software programme NVivo by Author 1. Open coding involved reading the transcripts line by line to identify and develop ideas, themes and issues from the data (20). A list of codes and sub codes was developed around dominant themes, for example, “help seeking”, “ambivalence”, and “lifestyle choice”, to then form the basis of the thematic analysis of the interview and diary data. In the research team meetings that followed between Author 1 and 2, axial (secondary) codes were developed. This stage of data analysis involved making comparisons across the data, so that the final stage of selective coding could occur. Selective coding involved taking core themes and positioning these as key theoretical frameworks for analysis, and critically examining them against the wider literature (52). As the participants had written in pen and pencil, the paper diaries were manually coded using the same thematic codes as the interview transcripts.

## Results: *Habitus* and Embodied Routines

### Participant Characteristics and Completion Rates of Data Collection

The five women in this study who were identified as meeting characteristics of SE-AN had all previously received a diagnosis of anorexia nervosa from a health care professional, and self-reported 10 to 30 plus years of eating disorder practices. Over their life course, they had experienced different eating disorder and other mental health diagnoses, attempts at recovery, and relapses, ambivalence, and refusal to seek therapeutic help (although they sought help for other mental health and medical conditions). The results of the EDE found that four out of five met the criteria for an eating disorder at the time of the interview. As mentioned in the introduction, while Charlotte did not meet the criteria for an eating disorder when the EDE was conducted^1^, she revealed a long history of severe anorexia nervosa that impacted her daily functioning and multiple admissions to in-patient care, locally and internationally. For this reason, and her long-standing struggles with SE-AN, we included her in this analysis.

While the sub-group of women we examine in this paper is small and a limitation of this study, the data is rich. Thirteen semi-structured interviews, 5 EDE interviews, detailed observations, field notes, and four diaries (one of the women declined to participate in the diary writing phase) provide what anthropologists call “thick description”—rich, contextual data describing decades of living with—of the embodiment of—anorexia nervosa.

### Safety and Routine

Over time the women described how their eating and body practices came to structure their everyday worlds. Routines brought relief from emotional distress, formed a strategy for coping with daily challenges, a safety net to fall back on and a familiar space to come back to. Kelly, who had lived with anorexia nervosa for over 20 years and described herself as a “healthy anorexic” (26), commented that over time “I think it gets easier and that’s one of the hardest things about it - is it gets so easy, it’s so natural”. Charlotte described how powerful the pull to disordered eating was even when on the road to recovery: “when everything else is crazy in your life it’s something to hold on to. It’s like a life ring”. She further explained, “something big will happen in your life … and it’s all feeling too overwhelming. You can actually make a conscious decision to turn around and find that life way, because you need something to hold on to and you know that it’s helped in the past”. Her disordered eating practices are thus positioned as the logical solution to a challenging situation because of the safety and familiarity that the structure of SE-AN provides.

Just 6 months before our first meeting, Sarah (28) had discharged herself from in-patient care for weight restoration. Physically fragile and reliant on her sister for care, Sarah remained steadfast in her wish not to give up her eating practices. She described the difficulty in trying to re-engage with social circles and study while maintaining her eating disorder routines. An evening education course caused great anxiety because it disrupted her evening eating practices. She would often cancel these classes, stating: “It’s such a relief to my system when I cancel. I know my routine will remain intact. I take real comfort in it”. Sarah’s routine for the past 7 years has been to only eat at night and vomit that meal up afterward. It is the same meal each day and she has rules and rituals which guide her. She chooses food to eat which she knows will be easier and less painful to expunge from her body, prioritizing “a practical sort of selection of foods” which are greasy and soft. There are also rules around eating the lowest calorie food first, preparing the food in the same order every time and only allowing “the food to remain in my body” for a set amount of time.

The women in our study spoke of the safety in their home spaces (in which most of the interviews took place) where they performed the majority of their eating and exercise routines. Sarah dreaded being out in public, writing in her diary: “I don’t want my physicality on display. I don’t want people to see me. I want to be hidden, private, protected”. Sarah said she only consumed water when in public and hadn’t eaten outside her home in 7 years. When the participants’ followed their strict eating and body routines, they spoke of it offering them some safety and protection when entering public spaces.

Eli, in her study of people with eating disorders in Israel, noted that for her study participants, “to engage in eating disordered practice was to transform any space, temporarily, into one’s own world” [(28), p. 481]. For Michelle, 27 years of age, “sticking to the regimes that I stick to, it seems to be the only time that I do feel okay about myself” and that “it makes me feel better being in my own skin”, especially when under “surveillance” in public and eating with friends and family. There are gendered and moralized norms in Australian culture about eating in public, and for women in particular, what and how much they eat is highly scrutinized and tied to stereotypes of femininity and health status. Participants inhabited and incorporated these cultural norms more intensely – eating a minimal amount or not at all—as a way of feeling safe in public spaces. By following cultural expectations of a gendered *habitus*, Michelle and others were able to more safely engage in relationships and the social world.

For Lorraine, at 52 years of age, who was diagnosed with anorexia nervosa at age 30 and reported having lived with it (and at some points bulimia nervosa) for over 30 years, her practices had become “a script that you just can’t shake free of”, which felt “safe” and offered “comfort”. Lorraine lived with her husband in a beachside suburb and worked part time in the public service, recently stepping down from a leadership position due to worsening mental health. She described seeing psychiatrists for anorexia nervosa on and off from age 30 but not wanting to “give it up”. However, at age 50, Lorraine reached out for help, describing her physical health worsening to the point of attempting suicide 6 months previous to the interview. Lorraine stated, “I had no quality of life left” and after the unsuccessful suicide attempt, she decided to engage with psychiatric therapy once again.

Like many people with anorexia nervosa, Lorraine was well aware of the damage that SE-AN was having on her health as she aged (fractures, low bone density, nausea). Prior to our meetings, she had put on some weight (an increase to 46 kg) in an attempt to improve her physical health and quality of life. She felt unsure about these weight changes, telling us that after decades of using anorexia nervosa to navigate the world, “you feel safe if you know what to expect if you stay on this sort of a routine”. As the eldest participant in this study, the embodied impacts of SE-AN were striking in Lorraine’s presentation and experiential accounts, and improving her physical health and quality of life—but not giving up the anorexia nervosa—was the primary motivation for her recent engagement with psychiatric services.

### Sociocultural Elements Which Support and Sustain People’s Eating Disorder Practices

In a previous paper, we have described how the women in this study were acutely aware that they lived in a culture that celebrated and equated good health with thinness and restriction (26). This earlier paper explored how the women capitalized on popular health aspects of their *habitus* (such as detox diets, yoga, veganism, and, more recently, fasting diets) and incorporated them into their disordered eating practices and routines, allowing them to engage with dominant gender ideologies of “healthy lifestyles” and “self-care”. Positioning their own practices as acts of care and health therefore negated their need for therapeutic care.

Participants sometimes joked about how they shared similar eating and body practices, aspirations, and ideals with those around them who did not have an eating disorder diagnosis. In interviews the women often compared their eating and exercise practices to their friends and family who were overweight or who were regularly on fad diets as justification for rejecting clinical labels of “illness” and “disorder”.

Michelle’s interaction with her trainer at the gym highlights this positioning of what may be considered by clinicians as eating disorder behaviors, but in sport and gym settings are an admirable demonstration of fitness, control and health:

I was at the gym the other day and my PT [Personal Trainer] was telling me that she’d been to this weight lifting conference … she talks about, you know “I have [to] really balance my carbs because if I don’t get enough carbs then my mood crashes and all this kind of stuff” … like what the hell? But there was this dude there who was talking about his training and was saying that he would deliberately stay up late and deprive himself of sleep because he’d be burning more calories. That’s like putting your body through hell to achieve a certain body shape … I did that and I was told I was sick.

LaMarre and Rice (54) examine how women with eating disorders are faced with navigating prescriptions for recovery in a sociocultural context that privileges some bodies and food-related behaviors over others. For example, they argue “following from a ‘war on obesity’ over the past two decades, fatness is stigmatized, equated with laziness, ill health, ugliness and a lack of restraint and will power” [(54), p. 138]. In her ambivalence toward recovery, Charlotte struggled with resisting these cultural ideals and gendered norms, highlighting that women with anorexia nervosa “continually negotiate culturally dominant understandings of subjectivity, embodiment and health” and in “adopting and disrupting” these norms, demonstrate their active role in their disordered eating [(55), p. 395]. Reflecting on how she is entangled with these cultural norms Charlotte stated, “we’ve got a whole society that advocates to have self-control, to be disciplined, to have restraint, you know these are all qualities that we advocate for people to have and this is just one area [anorexia nervosa] that you can put them into but also fits with the societal ideals of you know the slender, waif-like girlfriend”.

Toward the end of our data collection, when Authors 1 and 2 visited Charlotte’s house to collect her research diary, Charlotte informed us that she had finished and passed a Personal Training course, most of which occurred over the eight week diary writing phase. The diary documents how the Personal Training course became part of her search for a new routine, a way to remain thin and healthy and keep her eating disorder thoughts at bay. Charlotte had “never been much of an exerciser”, preferring to restrict her food intake to maintain a low weight, and was enjoying the new sensation of feeling “strong”. However, she increasingly struggled with being “triggered” during the course because the central focus of the lessons often ended up being about weight loss and appearance rather than a holistic approach to health and wellbeing. Charlotte then began restricting her diet again, stating she was becoming more “rigid” and “wanting to lose weight”, and soon found herself spiralling down the “rabbit hole” once again. In the end, Charlotte decided to put her Personal Trainer ambitions on hold.

The sociocultural *habitus* described above support disordered eating to such an extent that despite the harm caused by long term anorexia nervosa practices, the women transformed this endurance into a moral marker of success.

### “It’s the Glue” That Holds Everything Together

*Would you be reluctant to have to change your food/exercise practices?* This was a central question that each participant was asked and which often resulted in a strong response. The women described not having their eating practices as: “a big void in my life”, or like “losing the ability to breathe”. Kelly responded: “I’m always going to be anorexic, I just have to be able to manage it”, and Lorraine “couldn’t imagine not thinking about it and acting like I do and doing the things I do … yeah, it’s hard as it plays such a big role in my life and [my husband’s]”. Michelle stated her anorexia nervosa was “the one thing that could almost ground me”, and “it became a very tangible thing that I could say, this is my thing, and no one can touch it”. Michelle became pregnant during the project, and she viewed her weight gain and increased calorie intake as a temporary measure for the short period of time when her body “was not her own”. Following the birth of her baby her goal was to return to her restrictive eating practices and to lose the weight she had gained.

Despite the serious impacts on their health, relationships, and general wellbeing over many years, the women in our project were studying, held down jobs, went on holidays, and were parents, partners, church-goers, and supportive friends. They were highly skilled in managing their eating disorders. It was something that they always came back to, as Kelly described—“it’s the glue”, that became a “structuring structure” (43) to their everyday worlds. By her late 20s, Michelle had completed an honours degree at university, started a family and worked as a peer support worker at a mental health non-governmental organization (NGO). She had also been “in and out of psych hospitals since [her] early teens”, and explained that when she notices herself becoming overwhelmed and spiralling emotionally, she contacts her psychiatrist and asks to be admitted to hospital. When she is discharged the desire for anorexia nervosa has not receded, and she returns to her disordered eating routines. In-patient care had become a necessary and critical tool for Michelle to keep her well enough to continue maintaining her eating and body practices, to go to work, study, and have relationships. However, it was never her intention to stop her practices and despite many near death experiences she explained: “it’s like 90% of the time it’s just, whatever, like this is just what I’m doing”.

Lorraine explained that anorexia nervosa for her is “a lifestyle [in which] everything revolves around not eating or exercise or ‘when can I do this’ and ‘how much did I have here’, and weighing yourself and ‘how else can I lose weight now’?”. “Everything else”, she says “is organized around it”. Kelly similarly manages and plans her practices, or as she refers to them her “binge ups” and “starve downs”, around everyday life circumstances. For example, she is aware that people will treat her coldly at a party if she appears too thin, so she will do things to “plump” herself up in preparation. As she has aged her practices have taken a toll on her body and Kelly has adapted to “starving” more “healthily”. Her low blood pressure meant that she had to restrict high intensity forms of exercise, so Kelly took up yoga as a weight maintenance tool instead. She stated that with yoga “I’m allowed to be skinny, and people will say *how are you so muscly and thin?* I do yoga”. For these women, their eating disorder practices are normalized and conform to the structures and practices of a dominant, health focused *habitus*.

## Discussion: Se-An is Profoundly Embodied

In a recent commentary on the self in anorexia nervosa, Aminato et al. (56) outline a theoretical model of eating disorders in which they suggest that the self represents the organizing function of the mind. Referencing and following Hilda Bruch’s earlier work (57), these authors argue that “deficits in the self are the basis of eating disorder psychopathology, thus establishing anorexia as a disorder of the self” [(56), p. 849]. Explicitly drawing upon Cartesian metaphors of mind and body, a separation ensues between the person’s ability to recognize bodily sensations (such as hunger) and bodies become objectified as a “mere object”. In this theoretical model, the self “represents the organizing function of the mind that when disturbed, will lead to and maintain the disorder” [(56), p. 849], and the body sits as a passive backdrop.

As we have argued above, this clinical narrative of self is premised on western philosophical dualisms that split and privilege minds over bodies, insides over outsides. In their study of a group of women who had recovered from anorexia nervosa, Dawson et al. (13) found that during the illness phase, their participants (who had an average length of illness of 15.5 years) internalized anorexia nervosa and were unable to externalize the illness. As in our study, their participants’ perceived anorexia nervosa as “impossible to escape, all-consuming and chronic” (ibid). Recovery [for Dawson (13)] is understood as “externalization of anorexia”, or removing anorexia nervosa from the body, putting it outside the body. These culturally and historically constructed “[dualist] metaphors permeate the clinical field, shaping our own understandings and therapeutic practices” [(15), p. 2]. The dominant understanding of anorexia nervosa is thus as “a disorder of the self” (56) and “the self” is frequently the target of interventions for anorexia nervosa (16, 58, 59). However, in exploring recovery narratives, LaMarre and Rice [(54), p. 137] found a great variance in people’s understandings and experiences, with many rejecting the clinical ideas “that recovery entails an overcoming and divorcing of self from eating disorder”. Indeed, many of the participants in our study rejected the labelling of their practices as “illness” or “disorder”, and thus were ambivalent to the proposition that what they were experiencing could be cured or separated from their selves and social worlds.

Taking a different approach, social anthropologists understand “the self as culturally constituted” [(30), p. 5]. A full review of this large body of anthropological scholarship on “the self” is outside the scope of this paper, but the key point is that abstract and disembodied concepts of the self are “held to be outside of time, outside of space, outside of culture and outside of gender” [(60), p. 480]. If we approach SE-AN as the embodiment of cultural practices (situating the self in embodied, cultural contexts), we illuminate “generative schemes and bodily dispositions” that structure and give meaning to people’s everyday worlds. Embodiment is a “methodological standpoint in which bodily experience is understood to be the existential ground of *culture and self*” [(42), p. 269, our emphasis]. For people in our study, this generative scheme was not pathological, but a normative, culturally legitimated *habitus*.

To divorce oneself from anorexia nervosa would be to ask participants to not only give up the protective and productive aspects of anorexia nervosa, but also to step outside of culture. While aspects of safety and identity have been documented in eating disorder experiences (14, 24–26, 28, 61–63), framing these experiences through the concept of embodiment broadens how we understand people’s experiences and takes us beyond acultural selves. Anthropologist Karin Eli (28), for example, situates the individual experiences of anorexia nervosa within the concept of “social suffering”, in which suffering is understood to be “produced relationally, taking shape within networks” from the individual to the political (p. 478). The participants in Eli’s study described their daily disordered practices as “surviving”, as a way of being-in-the-world that kept them safe from distress caused by interpersonal relationships and structural pressures (such as caring for a family member with a disability and living in poverty). This approach highlights how the self cannot be separated from anorexia nervosa, as the embodiment of eating disorder practices is an individual’s response to a much more complex experience of “social suffering” (28). Here, the individual “self” and social structures are relational (not in opposition).

In supporting Eli’s arguments, we similarly found that social suffering was a significant component of people’s *habitus* in disordered eating. Charlotte’s embodiment of anorexia nervosa enabled her to keep safe from the dangers she had experienced as a child. Michelle described the same practices which led her to psychiatric in-patient care many times also helping her to stay alive. She said “sticking to the regime that I have to stick to becomes so overwhelming. But then at the same time it’s just such an enticing path to go down, and I’ll go down it over and over again … each time I put on weight, so I’m straight down the same path”. These pre-worn paths are shaped by an embodied distress which formed a map for being-in-the-world (30). Like Eli’s participants, the women’s practices went beyond an “expression of a sufferer’s distress, but as the manifestation of the broader social and structural configurations that brought this distress into being” [(28), p. 479]:

embodied distress is not simply relegated to the individual sufferer, but is understood as emerging from and implicating structural constraints and the difficult and sometimes oppressive bonds of social being. Rather than representing maladaptive, idiosyncratic coping mechanisms, when contextualized in the participants’ greater narratives, eating disordered practices emerge as grounded in a “logic of practice” [(28, 64), p. 489].

In placing embodiment as a framing approach to SE-AN, the body is not seen as a passive object, but plays an active role in how people inhabit and phenomenologically experience their worlds. The body is not inert or secondary to cognitive processes; participants in our study were highly attuned to bodily sensations, using their bodies as gauges for being-in-the-world. Over many years of what she described as “trial and error”, Kelly used feelings of hunger as a barometer to titrate her moods and energy levels:

You know, … when you don’t eat a lot you know how food affects you. You know what foods are going to do what to you and you suddenly know what you need so that you won’t need other foods … I find when I’m not eating, if I’ve gone for periods of time without eating I get really excited and hypo and I buzz around and get really lively … I get excited about being so light.

At 40, Kelly used her hunger as a way to settle into what Wright suggests is “a kind of working anorexia, not careering toward death, but [still] disciplined, self-contained” [(65), p. 172]. Her being-in-the-world was far from disembodied or a “deficit of self”, it was profoundly lived and felt in and through her body. Through her continual and fine-tuned awareness of embodiment, Kelly reveals the key relations between embodied selves and the world.

It may seem anathema to suggest that there is a “logic of practice” to practices which have historically (and continue to be) labelled as “illogical” and pathological. For the women we interviewed (and as many other anthropological analyses of eating disorders also describe), there is a clear cultural logic to eating disorder practices. This logic, as Csordas argues [(30), p. 12], is drawn from the conditions of *habitus*:

The *habitus* is the universalizing mediation which causes an individual agent’s practices, without either explicit reason or signifying intent, to be none the less “sensible” and “reasonable” … the structure which has produced [the *habitus*] governs practice, not by the processes of a mechanical determinism, but through the mediation of the orientations and limits it assigns to the *habitus’* operations in invention. As an acquired system of generative schemes objectively adjusted to the particular conditions in which it is constituted, the *habitus* engenders all the thoughts, all the perceptions, and all the actions consistent with those conditions, and no others [(43), p. 79–95].

The *habitus* is thus a “structuring structure”—a “socialized subjectivity which gives rise to and serves as the classificatory basis for individual and collective practices” [(66), p. 585]. These practices are culturally informed and shaped, and embodied as seemingly “naturalized” ways of thinking, feeling, acting and classifying the social world and [a person’s] location within it [(66), p. 586]. For our participants, SE-AN was their embodied *habitus*, their “structuring structure” of their being-in-the-world and could not be separated from their sense of self. This is a position that the Australian author Fiona Wright captures so eloquently in her biographical account of years of anorexia nervosa: “I’m finally accepting that my illness is my normal, that I have to find a way to dwell within and alongside it” [(67), p. 17].

### How Does an Embodiment Paradigm Inform Quality of Life Approaches to SE-AN?

While views vary on treatment approaches to SE-AN, the narratives of the women in this study contribute to a growing number of questions about treatment expectations, models, and outcomes. If SE-AN is central to this sub-groups’ embodied experiences of being-in-the-world, is it ethical to focus on cure and recovery over quality of life? If recovery is unrealistic and unachievable for this group, how might the goals of treatment be shifted? What are the best models of care to support people with SE-AN?

As discussed in the introduction, there has been a move in eating disorder treatment models for people with SE-AN to include more holistic approaches that focus on improving quality of life rather than understanding recovery only through the medical model of absence of illness. Touyz and Hay [(6), p. 2] suggest “we need to rethink our treatment strategies by drawing upon the patient’s strengths and competencies rather than merely paying attention to what is “wrong” with them. Undertaking treatment with a poorly motivated, chronically ill patient where loneliness, despair and an empty sense of self prevail, poses unique challenges for clinicians”.

Placing people who have lived with anorexia nervosa for most of their adult lives into standard recovery pathways poses a number of serious harms. People who are fully committed to SE-AN may feel guilty for taking up costly resources for more newly diagnosed and younger patients, or may feel undue pressure to please others by “getting better”. Both run a risk of instilling guilt, creating unrealistic pressures to achieve, and compounding treatment failure. Recognizing the unintended consequences of asking too much or pushing too hard doesn’t mean giving up (49), it means refocusing treatment toward harm minimization and quality of life. This involves letting go of clinical expectations to restore weight and minimize clinical symptoms, and focusing on the best ways to support people with SE-AN to have a quality of life. Westmoreland and Mehler [(49), p. 316] suggest that the goal of a harm reduction model for people with SE-AN is:

to assist patients in getting to a reasonable level of functioning that they can then maintain, rather than subjecting them to a full course of treatment, which usually involves a prolonged hospital stay to achieve restoration of ideal body weight. Candidates for this form of treatment are those who have endured multiple previous eating disorder treatments with minimal success, and those for whom full weight restoration has not been sustainable. Patients who undergo a “harm reduction” model of treatment are usually managed as outpatients. They are allowed to remain at a weight that is below their ideal body weight range, but one that is sufficient to enable them to have a reasonable quality of life, even if they cannot work or be fully independent.

Others have outlined how this shift in focus to harm minimization and quality of life includes treatment which prioritizes remaining hopeful, finding meaning and purpose in life, focusing on abilities over disabilities and not setting unrealistic goals of complete symptom resolution (8, 62). If, as we have suggested, SE-AN is seen as an embodied being-in-the-world that structures the everyday through one’s *habitus*, treatment that works within and with these practices may offer more hope than current approaches that seek to untether people from their worlds.

## Conclusion

In this paper, we have argued that the paradigm of embodiment helps to reframe an understanding of SE-AN, thereby allowing treatment approaches to emphasize improved quality of life over full recovery (in medical terms). Embodiment recognizes the protective and productive aspects of the person’s eating disorder practices (safety, comfort, structure) and that eating disorder experiences are supported by and produced in relation to sociocultural contexts. Embodiment is thus not just an individual’s pathological reaction to trauma, but a response to a much more complex experience of “social suffering” (28, 62), which informs their understanding of how to be in the world and how to get on in the world.

Approaching SE-AN through a paradigm of embodiment has important implications for therapeutic models that attempt to move anorexia nervosa away from the body and separate it from the self in order to achieve recovery. The medical model trajectory of recovery is based on a clinical narrative that “dualistically separates anorexia nervosa as an illness from its antithetical state of recovery, whereby the person returns to some premorbid state of normality” [(15), p. 2]. This asserts the clinical narrative as the person’s reality, rather than other ways in which they understand their everyday worlds and practices. We have argued that separating everyday bodily experiences—literally disembodying anorexia nervosa—is more than the loss of an identity, it would dismantle participants’ sense of being-in-the-world. Understanding how SE-AN is itself an embodied structure that structures every aspect of *habitus*, helps us to understand how disordered eating practices contribute to (and simultaneously) impede their quality of life, the fear of living differently [or as Lavis suggests “tasting other ways to live” (62)], and the safety that embodied routines bring. This points to why people persist with such severe and medically dangerous body and eating practices for years and decades. The implication for patient care is that opportunities for harm minimization and improving a person’s quality of life may be overlooked because their experiences do not fit the clinical narrative.

## Ethics Statement

The studies involving human participants were reviewed and approved by University of Adelaide Human Research Ethics Committee (H-2012-069) and Southern Adelaide Clinical Human Research Ethics Committee (SAC HREC EC00188). The patients/participants provided their written informed consent to participate in this study. Written informed consent was obtained from the individual(s) for the publication of any potentially identifiable images or data included in this article.

## Author Contributions

MW led the design of the study with input from CM and PG. CM and MW conducted the research and analyzed the data. CM and MW wrote the manuscript with revisions from PG.

## Conflict of Interest

The authors declare that the research was conducted in the absence of any commercial or financial relationships that could be construed as a potential conflict of interest.

## Footnotes

1 While severity ratings scored high for many of the items in Charlotte’s EDE (e.g., fear of weight gain, feelings of fatness, fear of losing control over eating, preoccupation with shape and weight) indicating presence of psychopathology of anorexia nervosa, the majority of Charlotte’s frequency data scores (e.g., patterns of eating) did not. In addition, Charlotte was not weighed, opting to tell the interviewer her weight which resulted in a BMI of 19. It is important to note that the EDE only captures behavioral features of eating disorders within a specific time frame (1–3 months), and Charlotte was by her own description, in a “better place” during the EDE.
